# Deep Learning Super-Resolution Technique Based on Magnetic Resonance Imaging for Application of Image-Guided Diagnosis and Surgery of Trigeminal Neuralgia

**DOI:** 10.3390/life14030355

**Published:** 2024-03-07

**Authors:** Jun Ho Hwang, Chang Kyu Park, Seok Bin Kang, Man Kyu Choi, Won Hee Lee

**Affiliations:** 1 Department of Neurosurgery, Kyung Hee University Medical Center, Seoul 02447, Republic of Korea; kleenex0004@naver.com (J.H.H.); changcha@khu.ac.kr (C.K.P.); 2 Department of Neurosurgery, Kyung Hee University College of Medicine, Seoul 02447, Republic of Korea; 3 Department of Urology, National Police Hospital, Seoul 05715, Republic of Korea; popbomb@naver.com; 4 Department of Neurosurgery, School of Medicine, Inje University Busan Paik Hospital, 75 Bokji-ro, Busanjin-gu, Busan 47392, Republic of Korea

**Keywords:** artificial intelligence (AI), deep learning, super resolution (SR), magnetic resonance imaging (MRI), trigeminal neuralgia (TN)

## Abstract

This study aimed to implement a deep learning-based super-resolution (SR) technique that can assist in the diagnosis and surgery of trigeminal neuralgia (TN) using magnetic resonance imaging (MRI). Experimental methods applied SR to MRI data examined using five techniques, including T2-weighted imaging (T2WI), T1-weighted imaging (T1WI), contrast-enhancement T1WI (CE-T1WI), T2WI turbo spin–echo series volume isotropic turbo spin–echo acquisition (VISTA), and proton density (PD), in patients diagnosed with TN. The image quality was evaluated using the peak signal-to-noise ratio (PSNR) and structural similarity index (SSIM). High-quality reconstructed MRI images were assessed using the Leksell coordinate system in gamma knife radiosurgery (GKRS). The results showed that the PSNR and SSIM values achieved by SR were higher than those obtained by image postprocessing techniques, and the coordinates of the images reconstructed in the gamma plan showed no differences from those of the original images. Consequently, SR demonstrated remarkable effects in improving the image quality without discrepancies in the coordinate system, confirming its potential as a useful tool for the diagnosis and surgery of TN.

## 1. Introduction

Trigeminal neuralgia (TN) is a pain disorder resulting from pathological changes in the trigeminal nerve [1]. Pain caused by TN can severely restrict physical activity and lead to significant emotional problems, such as intense stress and depression, adversely affecting an individual’s basic daily life. Therefore, proactive treatment is essential. The initial treatment for TN usually begins with pharmacological therapy. If the response to medication is poor or adverse drug reactions occur, surgical options such as microvascular decompression (MVD) or gamma knife radiosurgery (GKRS) may be considered [2,3,4,5,6]. The choice of treatment methodology for TN involves a comprehensive consideration of the severity of pain, vascular anatomy, and patient age, with the primary goal of both approaches being to accurately distinguish the cause of TN and minimize damage to normal tissues [4,5,6]. MVD is primarily considered; however, if pain persists or significant postoperative complications occur, GKRS may be an option [4,5].

GKRS for TN involves a series of steps, including frame fixation, image acquisition and registration, treatment planning, and gamma radiation exposure. Computed tomography (CT) and magnetic resonance imaging (MRI) are used during the image acquisition and registration phases [7,8]. For GKRS of the TN, it is crucial to ascertain the precise location of the trigeminal nerve, where MRI, in particular, provides the necessary positional information for accurate targeting, thereby enabling safer surgical procedures [6]. In summary, the success of GKRS for TN depends on precise imaging studies, followed by accurate treatment planning and targeting, based on which an appropriate radiation dose is prescribed [8,9,10].

The use of MRI for diagnosis and surgery reflects ongoing improvements in image quality and advancements in examination techniques to enable the accurate diagnosis and treatment of diseases. In terms of image quality, various studies have reported the effectiveness of applying image postprocessing techniques, such as image filtration and algorithms, to efficiently remove noise generated during imaging examinations [11,12,13]. Reducing noise in image quality significantly aids in a clearer distinction between normal and pathological tissues and offers considerable benefits for diagnosis and treatment. MRI encompasses various methods from the perspective of examination techniques. Techniques such as T2-weighted imaging (T2WI), T1-weighted imaging (T1WI), and proton density (PD) imaging provide characteristic imaging information specific to diseases and greatly assist in diagnosis and treatment [14,15,16,17].

Although the aforementioned technologies provide useful indicators for the diagnosis and treatment of diseases, there are several considerations when applying them to the diagnosis and surgery of TN. One primary concern is the issue arising from the MRI examination time and sequences. Clinically, techniques are employed to reduce MRI examination times to minimize the quality degradation caused by patient movement. However, this can lead to another cause of image quality degradation, as it does not ensure sufficient time for the examined tissues to recover signals, which adversely affects the accurate targeting of GKRS. Furthermore, traditional postprocessing techniques aimed at improving image quality often intentionally distort the original image (ground truth, GT), and the degree of such distortion is not sufficiently compensated for by improving the image quality [18].

Artificial intelligence (AI), particularly super-resolution (SR) techniques, is a viable method for the diagnosis and surgery of TN. On MRI, the presence of surrounding vessels compressing the trigeminal nerve and the degeneration of the nerve itself are key radiological indicators of TN’s pathological state of TN, each possessing distinct texture components [1,2,3,4,19,20]. For the accurate diagnosis and treatment of TN, it is essential to represent the texture components of the trigeminal nerve and surrounding structures without distortion. Deep learning-based SR demonstrates remarkable performance in learning the characteristics of images and generating high-resolution images by estimating the detailed information of small parts of the image and implementing the overall pattern of the image at a higher resolution [19,20,21,22]. Thus, deep learning-based SR has a significant advantage in learning complex patterns from input data and reconstructing fine texture information, implying higher improvement in image quality and minimal distortion of the texture information of the trigeminal nerve compared to traditional methods.

The characteristics of SR can be particularly useful for GKRS in the treatment of TN. Distortion of the texture information of the trigeminal nerve is the primary cause of misalignment of coordinates in GKRS for TN. The ability of SR to minimize texture information distortion while ensuring high image quality can enhance the success rate of TN’s GKRS by accurately targeting the coordinates. Therefore, this study aimed to evaluate the improvement in the quality of trigeminal nerve MRI data when SR is applied and to determine whether the reconstructed MRI can be accurately utilized for the coordinates in TN’s GKRS, thus offering a practical approach for use in actual GKRS procedures.

## 2. Subject and Experimental Methods

### 2.1. Image Data

**Figure 1:**
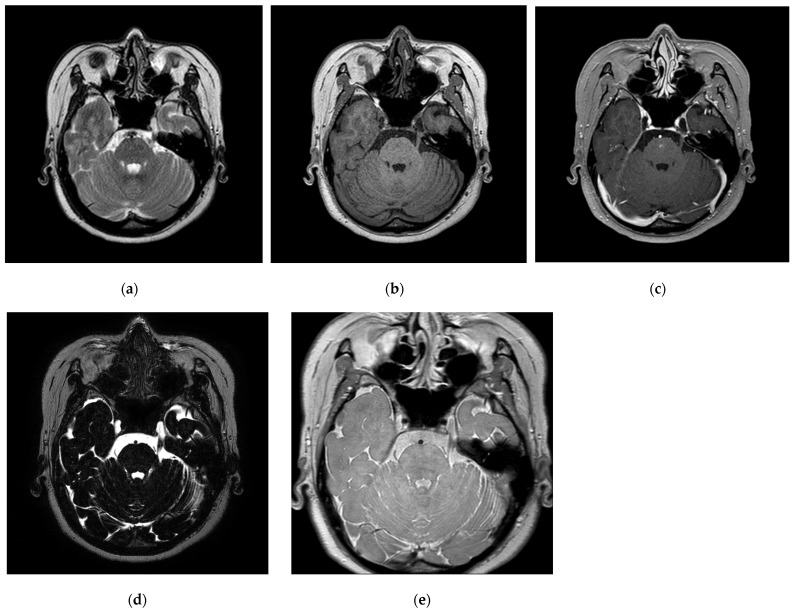
(**a**–**e**) Trigeminal nerve images (**a**): T2WI, (**b**): T1WI, (**c**): CE-T1WI, (**d**): VISTA, and (**e**): PD.

The data for this study were obtained from 31 individuals (eight males and 23 females) diagnosed with TN at our institution between October 2020 and July 2023, who underwent GKRS after experiencing recurrent and typical symptoms of TN despite pharmacological treatment and MVD surgery. For all GKRS procedures, a frame was fixed, and frame-based CT was performed to obtain coordinates for GKRS by co-registering the MRI and CT images. The target was the trigeminal nerve, with gamma radiation delivered using a 4 mm shot size. The MRI utilized internal imaging from our institution and was approved by the Institutional Review Board (IRB) for this retrospective study (IRB No. 2022-06-035). Figure 1 illustrates the imaging performed using a Philips Intra Achieva 3.0 tesla MRI scanner comprising five examination techniques: T2WI, T1WI, contrast-enhancement T1WI (CE-T1WI), T2WI turbo spin-echo sequence of volume isotropic turbo spin-echo acquisition (VISTA), and PD.

Table 1 presents the composition of the dataset and its parameters. The total dataset comprised 655 MRI data with a resolution of 512 × 512 and 16-bit processing. The detailed breakdown included 147 T2WI, 150 T1WI, 147 CE-T1WI, 116 VISTA, and 95 PD images. The examination parameters set to ensure the consistent image quality of the trigeminal nerve are as follows: for T2WI, spin echo (SE) sequence with a recovery time (TR) of 2500 msec, echo time (TE), and the number of excitations (NEX) of 1; for T1WI, gradient (GR) sequence with TR of 5 msec, TE of 2.5 msec, NEX of 1, and flip angle (FA) of 8; for CE-T1WI, GR sequence with TR of 5 msec, TE of 2.5 msec, NEX of 2, and FA of 8; for VISTA, SE sequence with TR of 2000 msec, TE of 316 msec, NEX of 1, and FA of 90; for PD, SE sequence with TR of 2000 msec, TE of 30 msec, NEX of 1, and FA of 90.

### 2.2. Dataset Preprocessing

After transferring the MRI data for TN to a picture archiving and communication system (PACS), the files were downloaded in digital imaging and communications in medicine (DICOM) format without compression. Subsequently, the DICOM data were converted into joint photographic expert group (JPEG) format images with 8-bit processing, and the input resolution was uniformly normalized to 256 × 256. During this process, MRI images containing moving artifacts, which were visually identified and thus not expected to improve image quality, were excluded.

### 2.3. SR Technique

**Figure 2:**
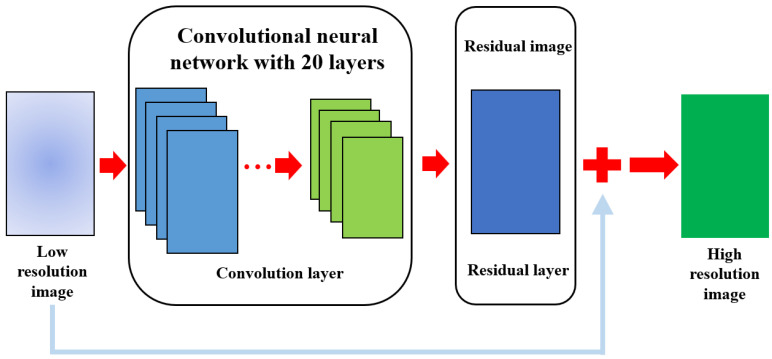
This Figure shows the proposed VDSR network for improving image quality. This illustrates the series of processes through which the input image passes through the convolution and residual layers, eventually producing a high-resolution image based on SR by summing it with the residual image.

SR implementation utilizes a deep super resolution (VDSR) network. Figure 2 shows a VDSR network that implements SR using low-resolution images, high-resolution images, and residual images.

In the implementation of SR using VDSR, if we denote the dataset as *N*, the low-resolution image as *X*, and the high-resolution image as *Y*, then the dataset is yielded by Formula (1):

(1) $${\{X^{\left(i\right)},Y^{\left(i\right)}\}\;}_{i=1}^{N}$$

The VDSR network aims to reconstruct a high-resolution image based on SR through training data. If we denote the network prediction as f and the reconstructed high-resolution image as $\bar{Y}$, then SR is yielded by Formula (2):

(2) $$\bar{Y}=f(X)$$

The VDSR defines the residual image R based on the fact that most components of the input and output images are similar. R is defined by Formula (3). In addition, if we denote the loss as L, then L follows Formula (4):

R = Y − X(3)

(4) $$L=\frac{1}{2}{\left|R-f(X)\right|}^{2}$$

Table 2 lists the hardware and software settings used to implement VDSR networks. The VDSR network was developed using MATLAB R2023a, utilizing image processing, deep learning, and parallel computing toolboxes. The computer specifications for deep learning included a Windows 11 Education operating system, a central processing unit (CPU) of Intel Core i9-12900KF (Intel, Santa Clara, CA, USA), and a GeForce RTX 3080 (NVIDIA, Santa Clara, CA, USA) graphics processing unit (GPU) at 12 GB.

### 2.4. Construction of Training Dataset

**Figure 3:**
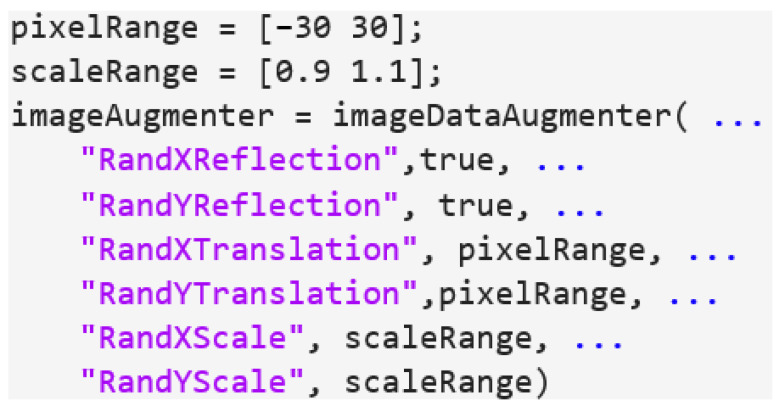
An example of the code used to augment the training dataset.

The GT images were first converted into the YCbCr color space and separated into luminance (Y channel) and chrominance (Cb and Cr channels). To create low-resolution sample images, the size of the Y channel was reduced, and the images were resized to their original size using bicubic interpolation. For this purpose, the training dataset was defined as pairs of up-sampled images with their corresponding residual images. These pairs were stored in the specified directories. To increase the number of training data, augmentations were applied by translating the images horizontally and vertically within a pixel range of (−30, 30) and scaling them within a range of (0.9, 1.1) to enlarge the dataset. The code for this procedure is shown in Figure 3.

### 2.5. Set Training Options

Table 3 lists network training options. Through iterative experimentation based on values known to be suitable for evaluating the performance of generalized models in various studies, an optimized set of training options was determined. Accordingly, the mini-batch options included a maximum of 100 epochs and a mini-batch size of 64, and the optimizer used was a stochastic gradient descent with momentum (SGDM), with a momentum of 0.9, an L2 regularization factor of 0.0001, a gradient clipping threshold of 0.01 (with the gradient threshold calculated using the L2 norm), an initial learning rate of 0.1, and a learning rate factor of 0.1, with the learning rate decreasing by a factor of 10 every 10 epochs.

### 2.6. Improving the Image Quality of the Trigeminal Nerve

The network layers were specified to facilitate the easy implementation of SR for medical imaging. The structure included an input layer, convolution layers, rectified linear unit (ReLU) layers, and an output layer, culminating in the implementation of a VDSR network. Subsequently, a VDSR network-based SR was applied to test the MRI data. Initially, the low-resolution images formed through sampling were resized to the size of the GT, and the image channels were converted to YCbCr. A bicubic interpolation was applied to each channel. Only the Y channel was isolated and passed through the VDSR network to produce a residual image. The components of the Y channel and output of the residual image were combined to obtain a high-resolution Y component. Finally, the high-resolution Y component was combined with the remaining chrominance channel components to output the SR-based high-resolution MRI data.

### 2.7. Evaluation of Image Quality and Coordinates of Surgical Planning

To assess image quality, two key metrics are commonly used: peak signal-to-noise ratio (PSNR) and structural similarity index measure (SSIM). To define the PSNR using the mean square error (MSE), two images were considered: Image 1 with pixel values $I_{1}$(*m*, *n*) and Image 2 with pixel value $I_{2}$(*m*, *n*), where *m* and *n* represent the number of rows and columns in the images, respectively. The MSE between these two images was calculated by Formula (5):

(5) $$MSE=\frac{1}{MN}\sum_{i=1}^{M}\sum_{j=1}^{N}{\left[I_{1}\left(m,\;n\right)-I_{2}\left(m,\;n\right)\right]}^{2}$$

When the maximum possible pixel value of an image is represented by R, the *PSNR* is defined by Formula (6):

(6) $$PSNR\;(db)=10\log_{10}\left(\frac{R^{2}}{MSE}\right)$$

To define the SSIM between two images, the dynamic range of the image is denoted as *L*, and the constants are $C_{1}$, $C_{2}$, $C_{3}$; these constants are defined by Formula (7):

(7) $$C_{1}={\left(0.01\times L\right)}^{2},\;C_{2}={\left(0.03\times L\right)}^{2},\;C_{3}=\frac{C_{2}}{2}$$

When comparing two images, referred to as Image 1 and Image 2 or *A* and *B*, respectively, and defining brightness as *l*, contrast as *c*, and structure as *s*, with μA and μB representing the mean luminance of images *A* and *B*, *σA* and *σB* as their standard deviations, and *σAB* as their covariance, the similarity in brightness *l*(*A*, *B*), contrast *c*(*A*, *B*), and structure *s*(*A*, *B*) can be defined by Formula (8):

(8) $$l(A,\;B)=\frac{2\mu A\mu B+C_{1}}{\mu^{2}A+\mu^{2}B+C_{1}\;},\;c(A,\;B)=\frac{2\sigma A\sigma B+C_{2}}{\sigma^{2}A+\sigma^{2}B+C_{2}\;},\;s(A,\;B)=\frac{\sigma AB+C_{3}}{\sigma A\sigma B+C_{3}\;}$$

Ultimately, considering the weights *α*, *β*, and *γ* for the similarities in luminance, contrast, and structure, the *SSIM* is defined by Formula (9):

(9) $$SSIM(A,\;B)={\left[l\left(A,\;B\right)\right]}^{\alpha }\times {\left[c\left(A,\;B\right)\right]}^{\beta }\times {\left[s\left(A,\;B\right)\right]}^{\gamma }$$

To ensure the reliability of the results, two neurosurgeons with over five years of AI research experience and one medical physicist evaluated the outcomes, and data with an SSIM of 1 between images were removed. The statistical significances of the calculated PSNR and SSIM for each technique were analyzed. The IBM SPSS version 23 program (IBM Co., New York, NY, USA) was used to conduct a paired *t*-test, and the statistical significance level was set at a 95% confidence interval, with *p* < 0.05 considered reliable. The coordinates for GKRS were evaluated by targeting the trigeminal nerve with a 4 mm shot using the dose algorithm of the tissue maximum ratio (TMR) 10 in the Leksell Gamma Plan 10.1 (Elekta, Stockholm, Sweden). In addition, to identify changes in the texture composition during the reconstruction process, the composition of the image pixels was plotted as a three-dimensional set.

## 3. Results

### 3.1. Improving the Image Quality of the Trigeminal Nerve

Table 4 describes the layer configuration of the VDSR network, which was reconfigured to predict SR in medical imaging. In the input layer, the patch size for the input image data was set to 41 × 41 to enable patch operations for the 20 VDSR layers. Since VDSR utilizes the luminance component, the Y channel was specifically designated, resulting in final channel dimensions of 41 × 41 × 1. The convolution layer consists of 20 layers, and network learning was facilitated by the operation of 3 × 3 filters, each composed of 64 units, with each operation being activated in the ReLU layer. The output layer was replaced with a regression layer to estimate the error between the residual image and the network prediction.

**Figure 4:**
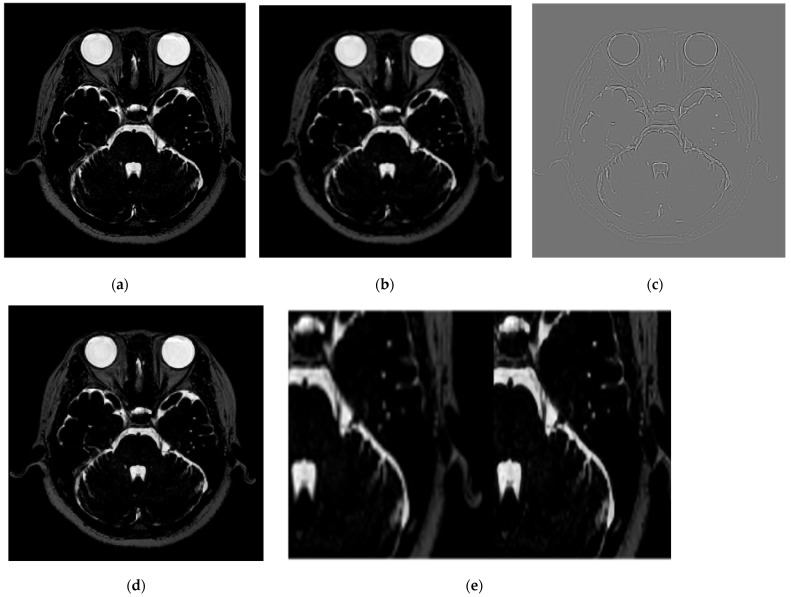
The process of obtaining high-resolution images through SR using the VISTA’s GT, low-resolution images, and residual images. (**a**) Represents the GT, (**b**) the low-resolution image, (**c**) the residual image, (**d**) the high-resolution image based on SR, and (**e**) an enlarged view for comparison of the trigeminal nerve between (**b**) (left) and (**d**) (right).

The test MRI images were converted into Y, Cb, and Cr channels, as shown in Figure 4. The Y channel of the MRI scanner was passed through the network to form a residual image. Finally, the high-resolution Y-channel component was combined with the remaining chrominance channels to output the MRI data with SR applied.

### 3.2. Evaluation of Image Quality and Coordinates of Surgical Planning

**Figure 5:**
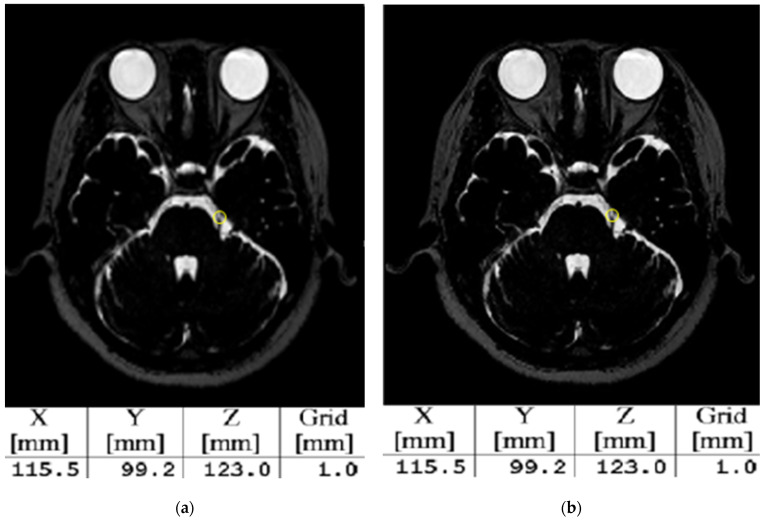
The coordinates system when high-resolution images based on SR were applied to the gamma plan. (**a**) Represents the GT and (**b**) shows a high-resolution image based on the SR. The yellow circle indicates a 4 mm shot targeted at the trigeminal nerve, and the X-, Y-, and Z-axis coordinates in (**b**) match those in (**a**).

Table 5 presents the results of evaluating the performance of SR by assessing the PSNR, SSIM, and coordinates of the trigeminal nerve. The image quality of MRI images subjected to SR showed the highest PSNR and SSIM values at a scale factor of two, and the results for PSNR and SSIM were statistically significant. Despite the differences in SSIM according to the scale factor, the GKRS coordinates between the GT- and SR-based data did not show any differences, as illustrated in Figure 5.

**Figure 6:**
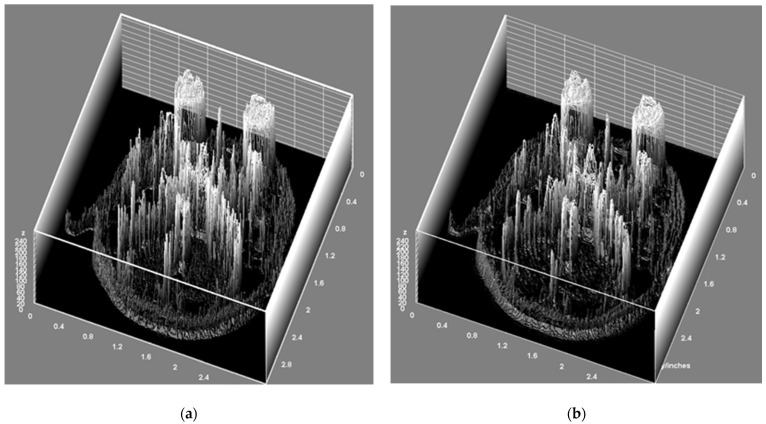
This Figure shows the texture components of the MRI data reconstructed using SR compared with those reconstructed using GT. (**a**) Represents the GT and (**b**) displays a high-resolution image based on the SR. This comparison suggests that there were no significant changes in texture composition when the MRI data were reconstructed using SR.

Figure 6 displays the original image alongside a high-resolution MRI image reconstructed based on SR to identify changes in texture composition.

## 4. Discussions

Various imaging techniques are used to accurately diagnose and treat diseases [23,24,25]. In particular, MRI provides valuable information for diagnosis and treatment by clearly distinguishing between normal tissues and lesions based on its high resolution in soft tissues [25]. The American Association of Physicists in Medicine (AAPM) offers several recommendations for maintaining optimal MRI [26,27,28]. Among these, a core aspect of the recommendations related to MRI resolution is the importance of quality control (QC) and the selection of appropriate examination parameters, emphasizing the need to efficiently manage noise occurring within images [27,28]. Hence, degradation in equipment performance and the selection of inappropriate examination parameters play a role in increasing the proportion of noise during signal and contrast formation in images, which can be a major cause of adverse effects in the diagnosis of diseases owing to decreased image quality [26,27,28].

Noise is not managed by QC, and the optimization of examination parameters occurs randomly within the images [28]. This noise is usually controlled through image postprocessing techniques such as filters, and various studies have been published on the usefulness of noise removal using postprocessing techniques [29,30,31]. However, this inevitably leads to a loss of image information. The problem is that the cost of image information loss is not compensated for by the expected improvement in quality, and in surgeries based on image guidance, such as GKRS, distortion due to loss of image information can be another cause of adverse surgical outcomes. This suggests that optimizing the image quality through traditional methods may no longer be the best approach, and there is a need to explore new methods that can address these issues. This study investigated the potential of applying deep learning-based SR as an image quality improvement strategy to address these existing issues and evaluated how the reconstructed images can be utilized in clinical situations requiring actual image guidance.

To improve the image quality of the TN, the network was selected by considering the depth of the layers and the amount of image data to be used. Deep learning calculates the patterns of complex image problems by feeding data into an artificial neural network (ANN). ANNs are typically composed of 8–201 layers, and various methods have been applied to minimize the time and computer resources required for pattern processing. Among the representative SR networks that reflect these characteristics are the super-resolution convolutional neural network (SRCNN) and VDSR. SRCNN, which utilizes a convolutional neural network (CNN), exhibits exceptional performance in enhancing image quality and has been proven to offer significantly better image quality improvement than traditional methods [32,33]. However, an SRCNN has a simple network structure, and it is challenging to apply a high learning rate, which limits its practical use [32]. As the depth of the layers in deep learning increases, so does the learning performance. However, SRCNN’s relatively simple design means it has lower learning performance, making it difficult to guarantee high image quality improvement. Additionally, network design does not allow for a high learning rate, leading to high computing resource costs [32,33]. Therefore, this study focused on the characteristics of VDSR, which exhibits high performance in improving image quality with its deep layers [32,33,34]. Compared with existing networks, VDSR is designed with 20 deep layers, enabling superior image quality improvement effects based on its excellent learning performance, allowing for the application of a high learning rate for smoother learning [32,34]. Although VDSR’s deep structure presents another limitation by complicating the learning process, this study aimed to implement single image super resolution (SISR) for a single trigeminal nerve in a single MRI slice among multiple MRI slices. Thus, VDSR was chosen as the network to improve image quality.

SR using the VDSR network was performed after a preprocessing step to efficiently train the MRI data. Specifically, this study focused on ensuring the seamless transmission of the Y channel to the network after converting the MRI data into YCbCr color channels, verifying that the up-sampled MRI and corresponding residual learning data pairs were correctly formed in the designated directory. This confirms that the trigeminal nerve MRI data can be reliably converted into YCbCr channels, and residual learning through the VDSR network can be effectively performed. Experiments using the test data also demonstrated the success of the network in outputting residual images, indicating the effective implementation of trigeminal nerve reconstruction based on SR. In terms of image quality improvement, VDSR showed superior effects compared with traditional methods. Compared with conventional image postprocessing techniques, the PSNR and SSIM values obtained using SR were significantly higher across all scale factors. A common issue with deep neural networks is the reduction in the size of feature maps through convolution layers, which potentially leads to the loss of pixel information that contains meaningful data. To prevent this, VDSR aims to preserve pixel information as much as possible through padding [32]. The high PSNR values across all scale factors reflected these characteristics. The SSIM values for images improved by SR were 0.86 for scale factors of 4 and 3, and 0.95 for a scale factor of 2. Notably, despite the differences in SSIM, the texture composition and gamma plan coordinates showed no differences between GT- and SR-based trigeminal nerve MRI [35]. In summary, compared to previous studies, this research highlights the ability to guarantee consistent results when applying the SR technique to trigeminal nerve MRI with different data beyond the training data, effectively improving PSNR and realizing accurate GKRS targeting as the reconstructed images’ X, Y, and Z coordinates match the GT- and SR-based images, despite differences in SSIM. This is the distinctive aspect of this study.

This study has limitations owing to the availability of MRI data and computer resource constraints, preventing the application of various methods that could aid in quality improvement. A primary limitation is the need for an SR network tailored for medical imaging. Most AI research emphasizes the importance of creating dedicated ANNs for specific purposes, with the majority of deep learning focusing on the classification and quality improvement of general images [35,36,37,38]. Although the VDSR used in this study shows generalized performance, its image quality improvement metrics tend to be somewhat lower than the PSNR improvement figures for general images [32,39]. In addition, the limited dataset used in the experiments may have led to overfitting. It is necessary to increase the available image data and consider overfitting when optimizing the network, specifically for SR in medical imaging. Second, ensuring the reliability of clinical applications is crucial. The GT- and SR-applied trigeminal nerve MRI data showed a similarity of 0.86, but it was unclear where differences in the reconstruction process occurred [35,40]. Therefore, even if there are no differences in the GKRS coordinates, the lack of objective evidence to claim accurate targeting of the GKRS is problematic if the key image information constituting the trigeminal nerve changes during the reconstruction process, regardless of the coordinates. SR capable of achieving SSIMs close to 100% of GT and AI, which can explain the results, are required. This necessitates checking the GKRS coordinates of the images reconstructed using various gamma plan versions and verifying the coordinates for multiple shots.

## 5. Conclusions

Despite the aforementioned limitations, this study significantly demonstrated the feasibility of applying SR to MRI images of the trigeminal nerve, quantitatively verifying its potential to improve image quality and its application in image-guided diagnosis and surgical environments. Furthermore, increasing the available datasets, improving SSIM, and verifying the GKRS coordinates of images reconstructed with various gamma plan versions represent additional research tasks crucial for accurately determining the targeting of the TN in GKRS and are worthy of future exploration.

## Figures and Tables

**Table 1:** Examination parameters.

MRI Methods	Image Resolution	Bit Processing (bits)	Number of Images (n)	Sequence	Recovery Time (msec)	Echo Time (msec)	Number of Excitations	Flip Angle (o)
T2WI	512 × 512	16	147	Spin echo	2500	100	1	-
T1WI			150	Gradient	5	2.5	1	8
CE-T1WI			147				2	
VISTA			116	Spin echo	2000	316	1	90
PD			95	Spin echo	2000	30	1	90

**Table 2:** Computer environment for SR implementation.

System	Computer Environment
Computer language	Matlab R2023a
Image processing	Image processing toolbox
Deep learning implementation	Deep learning toolbox
Dataset processing and computation	Parallel computing toolbox
Operating system	Windows 11 education
Central processing unit	Intel core i9-12900KF
Graphic processing unit	GeForce RTX 3080 12 GB

**Table 3:** Specifying learning parameters.

Options	Hyper Parameter	Value Setting
Mini batch options	Max epoch	100
	Mini batch size	64
Training options	Optimizer	Stochastic gradient descent with momentum
	Momentum	0.9
	L2 regularization	0.0001
	Gradient threshold	0.01
	Gradient threshold method	L2 norm
	Initial learning rate	0.1
	Learning rate factor	0.1
	Learn rate drop factor	10

**Table 4:** VDSR network.

Network	Layer Setting
Input layer	41 × 41 × 1 (Y channel)
Convolution layer	3 × 3 filter operation
ReLU layer	Activation function
Output layer	Replace with regression layer

**Table 5:** Image quality evaluation.

Dataset	Scale Factor	* Image Quality			
		* PSNR (db)		** SSIM	
		Bicubic Interpolation	Super Resolution	Bicubic Interpolation	Super Resolution
Internal MRI data	×2	31.5	32.6	0.9	0.95
	×3	26.3	26.4	0.84	0.86
	×4	26.5	27.6	0.83	0.86

* paired *t*-test, *, ** *p* < 0.05.

## Data Availability

The datasets used and/or analyzed during the current study are available from corresponding author on reasonable request.

## Abbreviations

AAPM, American association of physicists in medicine; AI, Artificial intelligence; ANN, Artificial neural network; CE-T1WI, Contrast enhancement T1-weighted imaging; CNN, Convolutional neural network; CPU, Central processing unit; CT, Computed tomography; DICOM, Digital imaging and communications in medicine; FA, Flip angle; GKRS, Gamma knife radiosurgery; GPU, Graphics processing unit; GR, Gradient; GT, Ground truth; IRB, Institutional review board; JPEG, Joint photographic experts group; MRI, Magnetic resonance imaging; MSE, Mean square error; MVD, Microvascular decompression; NEX, Number of excitations; PACS, Picture archiving and communication system; PD, Proton density; PSNR, Peak signal to noise ratio; QC, Quality control; ReLU, Rectified linear unit; SE, Spin echo; SGDM, Stochastic gradient descent with momentum; SISR, Single image super resolution; SR, Super resolution; SRCNN, Super resolution convolutional neural network; SSIM, Similarity index measure; TE, Echo time; TN, Trigeminal neuralgia; T1WI, T1-weighted imaging; TR, Recovery time; T2WI, T2-weighted imaging; VDSR, Very deep super resolution; VISTA, Volume isotropic turbo spin echo acquisition.
